# In Vitro Biological Activities of *Piper corcovadensis* C.DC.: Antioxidant, Antineoplastic‐Related and Antibacterial Effects

**DOI:** 10.1096/fj.202600527RRR

**Published:** 2026-06-05

**Authors:** Bruno Henrique Fontoura, Ellen Cristina Perin, Margarete Dulce Bagatini, Daiane Manica, Gilnei Bruno da Silva, Michelle Fernanda Faita Rodrigues, José Abramo Marchese, Solange Teresinha Carpes

**Affiliations:** ^1^ Department of Agronomy, Postgraduate Program in Agronomy (PPGAG) Universidade Tecnológica Federal Do Paraná Paraná PR Brazil; ^2^ Departamento de Tecnologia e Ciência de Alimentos Universidade Federal de Santa Maria Santa Maria RS Brazil; ^3^ Postgraduate Program in Biochemistry Universidade Federal de Santa Catarina Florianópolis SC Brazil; ^4^ Postgraduate Program in Biomedical Sciences Universidade Federal da Fronteira Sul Chapecó SC Brazil; ^5^ Multicentric Postgraduate Program in Biochemistry and Molecular Biology State University of Santa Catarina Lages SC Brazil; ^6^ Department of Chemistry, Postgraduate Program in Chemical and Biochemical Process Technology (PPGTP) Universidade Tecnológica Federal Do Paraná Curitiba PR Brazil

**Keywords:** inflammatory profile, oxidative stress, phenolic content, reactive species, SK‐MEL‐28 cells

## Abstract

Oxidative stress, caused by excessive reactive oxygen (ROS) and nitrogen species (NOX), is linked to degenerative, inflammatory, and cancer‐related conditions. *Piper corcovadensis*, a South American native plant, is recognized for its bioactive compounds. This study investigated the total phenolic content (TPC) by the Folin–Ciocalteu method, antioxidant activity (AA), cytotoxicity, and antimicrobial and anti‐inflammatory effects of its extract. AA was determined by the ABTS, DPPH, and FRAP assays. Cytotoxicity was evaluated on SK‐MEL‐28 melanoma cells through the MTT assay. Oxidative stress biomarkers (ROS, NOX), protein thiols (PSH), and non‐protein thiols (NPSH) and inflammatory mediators (NLRP3, *IL‐6*, *TNF*) were analyzed by RT‐qPCR at 50, 500, and 5000 μg mL^−1^. Antibacterial activity against *Bacillus subtilis, Escherichia coli, Listeria monocytogenes,* and *Salmonella Typhimurium* was assessed using MIC and MBC. The extract showed 11.64 mg GAE g^−1^ and AA of 33.15 mM Trolox g^−1^ (ABTS), and 21.27 mM Trolox g^−1^ (DPPH). At 500 and 5000 μg mL^−1^, it reduced SK‐MEL‐28 viability and decreased ROS, NOX, and PSH. At 5000 μg mL^−1^, it downregulated NLRP3 and *IL‐6* while upregulating *TNF*. MIC and MBC were > 5 mg mL^−1^ for all strains and showed no antibacterial activity at the tested concentrations. However, the extract of *Piper corcovadensis* exhibits significant in vitro bioactivity, particularly in antioxidant capacity, modulation of oxidative stress markers, and effects on melanoma cell viability.

## Graphical abstract

*Piper corcovadensis* extract is rich in bioactive compounds with antioxidant potential, capable of decreasing ROS levels; antitumor activity in cutaneous melanoma cells SK‐MEL‐28; and anti‐inflammatory activity, decreasing the expression of IL‐6 and NLPR3 genes and increasing TNF expression, without cytotoxicity to healthy peripheral blood cells (PBMCs). Besides, the extract showed antimicrobial activity against *Bacillus subtilis* , *Listeria monocytogenes* , *Escherichia coli* , and *Salmonella Typhimurium* .

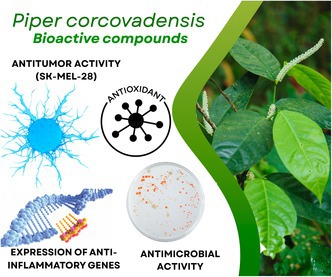

## 1 Introduction

Bioactive compounds derived from plants, such as *Piper corcovadensis* C.DC., a species native to South America, have shown promising therapeutic potential due to their antioxidant properties. Commonly known as João Brandinho, this plant is traditionally used by local communities to treat colds, flu, toothaches, and tuberculosis. Previous studies reported that its essential oil exhibits antioxidant, antimicrobial, and cytotoxic effects against tumor cell models [1]. Additionally, previous studies demonstrated the larvicidal effect of *P. corcovadensis* essential oil against *Aedes aegypti* L. [2]. However, the bioactive compounds present in the ethanol extract of this plant remain largely unexplored in terms of their therapeutic applications.

Oxidative stress plays a critical role in the onset and progression of diseases, including Alzheimer's, inflammation, atherosclerosis, and cancer, making antioxidants essential for maintaining cellular balance [3, 4]. Reactive oxygen species (ROS) are highly reactive molecules characterized by unpaired electrons or unstable chemical bonds [5]. Key examples include superoxide radicals (●O_2_^−^), singlet oxygen (^1^O_2_), hydroxyl radicals (●OH), and hydrogen peroxide (H_2_O_2_), as well as reactive nitrogen species (NOX), which exhibit even greater reactivity [6]. These radicals are natural byproducts of aerobic metabolism and play essential roles in physiological processes. Nevertheless, excessive accumulation can overwhelm endogenous antioxidant defense systems, leading to oxidative stress [7].

An imbalance in these systems can trigger oxidative damage at the cellular level, contributing to the development of degenerative diseases such as Parkinson's, Alzheimer's, cancer, and inflammatory disorders [3, 4, 8]. In particular, melanoma a highly aggressive form of skin cancer closely linked to oxidative stress and increased ROS production due to sun exposure and has been rising globally [9]. This malignancy is characterized by its high metastatic potential and significant mortality rates [8].

While conventional treatments such as chemotherapy, radiotherapy, and immunotherapy remain the primary strategies for combating cancer, plant‐derived bioactive compounds offer a promising alternative due to their antioxidant, anti‐inflammatory, and antimicrobial properties [9, 10].

In this context, the present study aimed to investigate oxidative stress‐related biochemical parameters, antioxidant activity, effects on melanoma cell viability, inflammatory markers, and antimicrobial activity of the ethanol extract from the leaves of *Piper corcovadensis* C.DC.

## 2 Materials and Methods

### 2.1 Chemical Reagents

Ethanol (99.5%) and Folin–Ciocalteu phenol reagent were purchased from Êxodo Científica Ltda. (Sumaré, SP, Brazil). 2,2‐Diphenyl‐1‐picrylhydrazyl (DPPH), 2,2′‐azino‐bis(3‐ethylbenzothiazoline‐6‐sulfonic acid) (ABTS), 2,4,6‐tris(2‐pyridyl)‐s‐triazine (TPTZ), and gallic acid were obtained from Sigma‐Aldrich Co. (St. Louis, MO, USA). Cell culture plates and flasks were sourced from Gibco (Thermo Fisher Scientific, Grand Island, NY, USA). Brain Heart Infusion (BHI) broth (IonLab, Araucária, PR, Brazil), Nutrient Agar (Himedia, Curitiba, PR, Brazil), chloramphenicol (Vetec Química Fina, Duque de Caxias, RJ, Brazil), and resazurin sodium salt (Sigma‐Aldrich Chemical Co., St. Louis, MO, USA) were also used in the study.

### 2.2 Plant Material

The leaves of *Piper corcovadensis* were collected in Rio Branco, AC, Brazil (7° 39′ 54′ S; 72° 39′ 1′ W; at 193 m altitude). The plant material was identified as *Piper corcovadensis* C. DC. and deposited in the herbarium of Midwest State University in Guarapuava, PR, Brazil, under the deposit code ARAUCA 1154.

### 2.3 Sample Processing and Extract Preparation

The plant leaves were dried in an air‐circulating oven (Mylabor, model SSD 11 L, Tatuapé, SP, Brazil) at 37°C ± 3°C until constant weight. After drying, the leaves were ground using a knife mill (Tecnal, model R‐TE‐650/A, Piracicaba, SP, Brazil) to obtain a fine powder with an approximate particle size of 30 mesh.

The extraction conditions were previously optimized based on the antioxidant potential of *Piper corcovadensis* leaves, considering solvent concentration, extraction temperature, and extraction time [11]. In that study, the data were analyzed using a generalized linear model, and a chemometric prediction model was developed from digital image analysis [11].

In the present study, the extract was obtained using 0.1 g of dried and ground *P. corcovadensis* leaves and an ethanol/water mixture (80:20, v/v) as the extraction solvent. The extraction was performed in a water bath at 70°C for 120 min. After extraction, the ethanol from the supernatant was removed using a rotary evaporator (Tecnal, model TE‐211, Piracicaba, SP, Brazil), and the residue was subsequently dried in a bench lyophilizer (Liobras, model L101, São Carlos, SP, Brazil) for 5 days to obtain a dry extract.

### 2.4 Total Phenolic Content (TPC) and Antioxidant Activity (AA)

TPC and AA were performed using a UV–Vis spectrophotometer (Model KASVI K37, Pinhais, PR, Brazil). TPC was quantified by the Folin–Ciocalteu method at 740 nm, and the results were expressed as mg GAE g^−1^ (gallic acid equivalent) [12].

AA was evaluated using three methods, including the ABTS^●+^ (2,2‐azino‐bis‐(3‐ethyl‐benzothiazoline‐6‐sulfonic acid)) assay, with UV readings at 734 nm and results expressed as μM TE g^−1^ (TE: Trolox equivalent) [13]. DPPH^●^ (2,2‐diphenyl‐1‐picrylhydrazyl) assay, readings at UV readings at 514 nm, and results expressed as μM TE g^−1^ [14]. Finally, the FRAP (ferric reducing antioxidant power) assay, using the TPTZ reagent (2,4,6‐tris(2‐pyridyl)‐s‐triazine), included UV readings at 595 nm and results expressed as mM Fe^2+^ g^−1^ [15].

### 2.5 Assessment of *P. corcovadensis* Extract's Effect on Cell Viability

#### 2.5.1 Melanoma Cell Culture

SK‐MEL‐28 cutaneous melanoma cells were obtained from the Cell Bank of Rio de Janeiro (BCRJ, Rio de Janeiro, Brazil). The cells were cultured following the protocol described previously [16], using basal culture medium DMEM (Dulbecco modified Eagle Medium) (Vitrocell, Campinas, SP, Brazil), supplemented with 10% fetal bovine serum and 1% antibiotic‐antifungal (penicillin and streptomycin).

#### 2.5.2 Non‐Tumoral Cell Culture

##### 2.5.2.1 Peripheral Blood Mononuclear Cells (PBMCs) Acquisition

PBMCs were used to evaluate the extract's cytotoxicity on non‐tumor cells. These cells were obtained by venipuncture from two healthy individuals, collected in EDTA tubes, and separated using the Ficoll‐Histopaque gradient method according to the Böyum protocol [17], with adaptations. After collection, the whole‐blood tubes were centrifuged at 3500 rpm for 15 min. The buffy coat, containing PBMCs, was extracted, diluted in saline (1:1), and gently layered over the Ficoll‐Histopaque layer in a conical tube. The system was then centrifuged at 1800 rpm for 30 min. The PBMC layer was collected, washed twice with 10 mL of saline, and centrifuged again at 1800 rpm for 5 min. When necessary, hemolytic buffer was used to eliminate remaining red blood cells.

The collection of PBMCs from donors was approved by the Human Ethics Committee of the Federal University of Fronteira Sul (UFFS, Campus Chapecó, SC, Brazil) under protocol number 8.162.593. Blood collection was performed only after all donors provided written consent.

##### 2.5.2.2 PBMCs Culture

PBMCs were cultured in the specific medium RPMI (Roswell Park Memorial Institute) (Vitrocell, Campinas, SP, Brazil), supplemented with 10% fetal bovine serum and 1% antibiotic‐antifungal (penicillin and streptomycin) under the required conditions, as described by Da Silva et al. [18].

#### 2.5.3 Treatment of Cells With *P. corcovadensis* Extract

The *P. corcovadensis* lyophilized extract was solubilized in a solution containing the cell culture medium (DMEM for SK‐MEL‐28 and RPMI for PBMCs) with DMSO (dimethyl sulfoxide) (final concentration of 0.2%) to prepare a master solution. Subsequently, the master solution was diluted to prepare working solutions at concentrations of 50, 500, and 5000 μg mL^−1^. Both cell types were then treated with these solutions for 24 h. The cells in the negative control group (CT) were exposed only to culture medium.

#### 2.5.4 SK‐MEL‐28 and PBMCs Viability Using the MTT Method

The cytotoxicity of *P. corcovadensis* extract in SK‐MEL‐28 and PBMCs was evaluated using MTT assay (3‐(4,5‐dimethyl‐2‐thiazolyl)‐2,5‐diphenyl‐2H‐tetrazolium bromide) (Sigma Aldrich, MO, USA) [19]. SK‐MEL‐28 (2.5 × 10^4^ cells/well) and PBMCs (1 × 10^5^ cells/well) were cultured in 96‐well plates and exposed to the treatments. After 24 h, the MTT reagent was added, and the mixture was incubated for 2 h at 37°C. Viable cells reduced MTT to formazan crystals (purple), which were then dissolved in DMSO. Absorbance was measured at 570 nm, using a microplate reader (Multiskan GO, Thermo Scientific).

#### 2.5.5 Cell Viability by Fluorescence Microscopy

Fluorescence analysis was performed as described previously [20]. SK‐MEL‐28 cells (1 × 10^4^) were seeded in 96‐well plates and incubated for 24 h. After incubation, 80 μL of HBSS (Hank's Balanced Salt Solution), 10 μL of 20 nM TMRE (tetramethylrhodamine ethyl ester), and 10 μL of 30 mM DAPI (4′,6‐diamidino‐2‐phenylindole) were added and incubated for 30 min. The wells were washed twice with 0.9% NaCl (saline solution) before fluorescence measurements. The excitation and emission wavelengths were set as follows: excitation at 550 nm and emission at 580 nm for TMRE; excitation at 358 nm and emission at 461 nm for DAPI. Fluorescence analysis was performed in triplicate. Images were captured using a fluorescence microscope (Nikon Eclipse TS2‐FL, 200× magnification) and processed for brightness and contrast using ImageJ software. Results were expressed as mitochondrial membrane potential (ΔΨm) for TMRE and fluorescence intensity (%) for DAPI, relative to the control.

#### 2.5.6 Cellular Biochemical Processes of Oxidative Stress

Oxidative stress parameters were evaluated by measuring intracellular reactive oxygen species (ROS), nitric oxide (NOx), total thiols (PSH), and non‐protein thiols (NPSH) in SK‐MEL‐28 cells (2.5 × 10^4^ cells in 96‐well plates). All analyses were performed in triplicate.

#### 2.5.7 Intracellular Reactive Oxygen Species (ROS)

Intercellular ROS levels were measured using a fluorometric intracellular ROS commercial kit (Sigma Aldrich, St. Luis, MO, USA), following the manufacturer's instructions. The fluorescence intensity, proportional to the amount of ROS, was measured at an excitation wavelength of 490 nm and an emission wavelength of 520 nm using a microplate reader (Varioskan LUX, Thermo Scientific, Waltham, MA, USA).

#### 2.5.8 Nitrite/Nitrate Determination

Nitrite and nitrate levels were quantified using the method described by Tatsch et al. [21] Griess reagent was incubated with the samples for 30 min at 37°C, thereby forming a diazonium salt. This salt then reacted with N‐(1‐naphthyl)‐ethylenediamine (NED), producing a purple azo dye. Absorbance was measured at 540 nm. Analyses were performed in triplicate, and results were expressed as a percentage (%) relative to the control.

#### 2.5.9 Total Thiols (PSH) and Non‐Protein Thiols (NPSH) Contents

Total and non‐protein thiols levels were determined based on the reduction of 5,5′‐dithiobis (2‐nitrobenzoic acid) (DTNB), with absorbance measured at 412 nm. For total thiol analysis, 40 μL of the sample was transferred to a 96‐well plate, followed by the addition of 200 μL of 1 M potassium phosphate buffer (PPB) at pH 6.8. After mixing, 20 μL of DTNB was added, and absorbance was measured immediately. For non‐protein thiols, the procedure was similar, but the samples were deproteinized with 10% trichloroacetic acid (TCA). Then, 30 μL of the supernatant was used for analysis, following the same procedure. Results were calculated using a cysteine standard curve and expressed in μmol L^−1^ [22].

### 2.6 Expression of Inflammation Markers

The expression levels of *NLPR3* (NLR Family Pyrin Domain Containing 3), *IL‐6* (Interleukin‐6), and *TNF* (Tumor Necrosis Factor) were assessed by real‐time quantitative reverse transcription polymerase chain reaction (RT‐qPCR) in SK‐MEL‐28 (1 × 10^6^ cells in a 6‐well plate). After cell culture and 24 h of extract treatment, total RNA was extracted using TRIzol reagent, and RNA purity and quality were evaluated spectrophotometrically (Thermo Scientific Varioskan LUX). Complementary DNA (cDNA) was synthesized from total RNA using a high‐capacity cDNA reverse transcription kit (Thermo Scientific, USA) with random primers. RT‐qPCR was performed using 6 μL of cDNA, 10 μL of PowerUp SYBR Green Master Mix (Applied Biosystems), and 2 μL of each primer (500 nM). The amplification protocol included an initial denaturation at 95°C for 5 min, followed by 60 cycles of denaturation at 95°C for 15 s and annealing/extension at 60°C for 60 s. The identity of the PCR products was confirmed by melting curve analysis. All experiments were conducted in triplicate. Gene expression levels were normalized against the housekeeping gene GAPDH (glyceraldehyde‐3‐phosphate dehydrogenase) using the ΔΔ^Ct^ method [23].

Primer sequences (5′–3′): GAPDH (F):

CTCCTCACAGTTGCCATGTA; (R): GTTGAGCACAGGGTACTTTATTG.

NLRP3 (F): CCATCGGCAAGACCAAGA; (R): ACAGGCTCAGAATGCTCATC.

IL‐6 (F): TCATCCCATAGCCCAGAGCA; (R): CTGGCATTTGTGGTTGGGTC.

TNF (F): CAGGCAGTCAGATCATCTTC; (R): GCTTGAGGGTTTGCTACAAC.

### 2.7 Antibacterial Activity

The minimum inhibitory concentration (MIC) and minimum bactericidal concentration (MBC) were determined according to the Clinical and Laboratory Standards Institute (CLSI) guidelines [24]. The bacterial strains used in the assay included *Salmonella Typhimurium* (ATCC 14028), *Escherichia coli* (ATCC 25922), *Bacillus subtilis* (ATCC 6054), and *Listeria monocytogenes* (ATCC 19111). A concentration of 1–2 × 10^5^ UFC/mL was obtained by adding 50 μL of the bacterial suspension to 50 mL of nutrient broth. The extract concentrations used in this analysis were 5, 2.5, 1.25, 0.63, 0.31, and 0.16 mg mL^−1^. In the microplate, the MIC was determined using 190 μL of inoculated nutrient broth and 10 μL of each extract. Chloramphenicol 0.12% was used as the positive control, ethanol as the negative control, and resazurin dye was employed to identify the positive wells. The plates were maintained for 24 h at 37°C. For the MBC test, an aliquot from each positive well in MIC was inoculated into a Petri dish containing nutrient agar and incubated for 24 h at 37°C. The MBC was defined as the lowest concentration at which no visible microbial growth was observed. The experiment was performed three times, and the results are presented in mg mL^−1^ of the extract.

### 2.8 Statistical Analysis

Statistical analyses were performed using GraphPad Prism 9 software. A one‐way analysis of variance (ANOVA) was applied to the data, with *p* < 0.05 set as the criterion for statistical significance. Significance levels were defined by *p*‐values *(*p* < 0.05), **(*p* < 0.01), ***(*p* < 0.001), and **** (*p* < 0.0001).

## 3 Results and Discussion

### 3.1 Evaluation of Phenolic Compounds and Antioxidant Potential

The values obtained for TPC and AA are available in Table 1. To evaluate the antioxidant potential, the ABTS, DPPH, and FRAP methods were employed. These methods are complementary, enabling quantification of antioxidant potential across a broad range of bioactive compounds [25].

**TABLE 1:** Total phenolic compounds (TPC) and antioxidant activity of *Piper corcovadensis C*. DC.

TPC (mg GAE g^−1^)	ABTS (mM TE g^−1^)	DPPH (mM TE g^−1^)	FRAP (mM Fe^+2^ g^−1^)
11.64 ± 0.044	33.15 ± 11.66	21.27 ± 0.535	13.47 ± 1.37

*Note:* GAE: Gallic acid equivalent; TE: Antioxidant capacity equivalent to Trolox.

The TPC was determined to be 11.64 ± 0.044 mg GAE g^−1^. The antioxidant potential of these compounds yields values of 33.15 ± 11.66 mM TE g^−1^ and 21.27 ± 0.0535 mM TE g^−1^ by the ABTS and DPPH assays, respectively. The form FRAP method resulted in a value of 13.47 ± mM Fe^2+^ g^−1^.

A previous study by Conde‐Hernández and Guerrero‐Beltrán [26] on *Piper auritum* reported TPC values ranging from 6.79 to 39.81 mg GAE g^−1^. According to the authors, this variation is influenced by the extraction method, with TPC levels increasing as the ethanol concentration in the ethanol/water (v/v) mixture increased. The values obtained in the present study agree with those of that study.

Similarly, research on *Piper chaba* reported a TPC of 5.02 ± 0.42 mg GAE g^−1^ and an antioxidant activity of 20.74 ± 2.80 mg ascorbic acid equivalent (AAE) g^−1^ in the ethanolic extract, measured by the DPPH method [27]. These values are comparable to those determined in this study, suggesting that levels found here are characteristic of plants from the *Piper* genus.

Phenolic compounds, including phenolic acids, flavonoids, and anthocyanins, are known for their potential role in disease prevention. Notably, these compounds have been associated with inhibiting tumor cell initiation and proliferation [28].

### 3.2 Effects of *Piper corcovadensis* Extract on Melanoma Cell Viability and Cytotoxicity in Non‐Tumoral Cells

**FIGURE 1:**
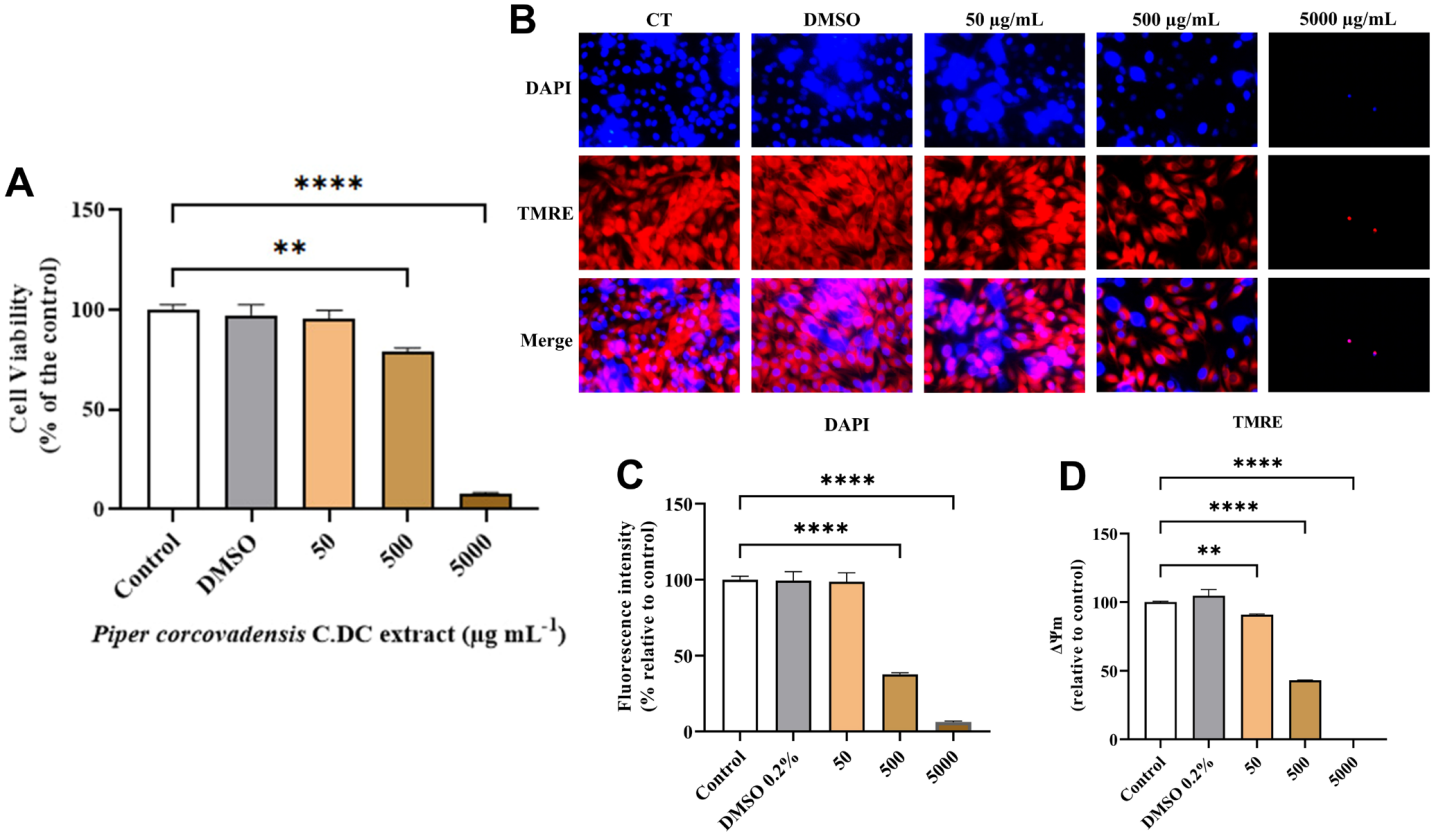
(A) Cell viability (%) in human cutaneous melanoma cells SK‐MEL‐28. (B) Fluorescence microscopy image showing the effect of *Piper corcovadensis* extract at different concentrations on SK‐MEL‐28 cells. (C) Fluorescence intensity (%) (DAPI) in the nuclei of SK‐MEL‐28 cells. (D) Mitochondrial membrane potential (ΔΨm) assessed by TMRE. Sample size for cell viability (*n*): 5. Sample size for cell fluorescence (*n*): 3. Significance levels were defined by *p*‐values **(*p* < 0.01), ****(*p* < 0.0001).

Cell viability in cutaneous melanoma cells (SK‐MEL‐28) was assessed 24 h after treatment with *Piper corcovadensis* C. DC. extract (Figure 1A). At a concentration of 50 μg mL^−1^, *Piper corcovadensis* did not reduce cell viability compared to the control. However, a significant decrease in cell viability was observed at 500 μg mL^−1^ (*p* < 0.001). At the highest concentration tested (5000 μg mL^−1^), cell viability was further reduced (*p* < 0.0001).

To support the MTT assay results, fluorescence analysis (Figure 1B) showed decreases in both nuclear fluorescence intensity (DAPI staining) and mitochondrial integrity (as indicated by TMRE staining) with increasing *concentrations of Piper corcovadensis*.

In Figure 1C, a significant reduction in DAPI fluorescence intensity was observed starting at 500 μg mL^−1^, with a marked decrease at 5000 μg mL^−1^ (*p* < 0.0001). Regarding mitochondrial membrane potential (ΔΨm), measured by TMRE (Figure 1D), a significant decrease was detected at 50 μg mL^−1^, with progressive reductions at higher concentrations, culminating in a complete loss of ΔΨm at 5000 μg mL^−1^ (*p* < 0.0001).

**FIGURE 2:**
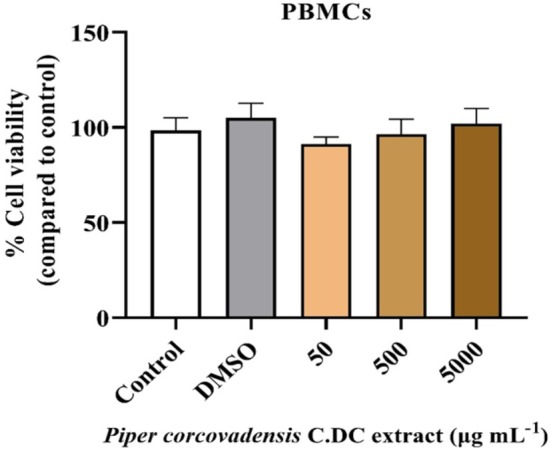
Cell viability of healthy peripheral blood cells (PBMCs) treated with *P. corcovadensis*. Sample size (*n*): 3.

Given the effects of *Piper corcovadensis* extract on melanoma cell viability, the non‐tumoral cells, PBMCs, were treated with the same concentrations as SK‐MEL‐28 for 24 h to assess a possible cytotoxic effect. Surprisingly, none of the tested concentrations reduced PBMC viability (Figure 2). These results suggest that, under the experimental conditions employed, the extract did not reduce PBMC viability at the tested concentrations, but did reduce melanoma cell viability.

In a previous study with *Piper corcovadensis* essential oil, a decrease in SK‐MEL‐28 cell viability was observed at concentrations of 62.5 and 250 μg mL^−1^, without cytotoxicity in PBMCs [1]. The main compounds of the essential oil were identified as sesquisabinene transhydrate (24.91%), *trans*‐caryophyllene (10.75%), *trans*‐β‐farnesene (5.22%), 14‐hydroxycaryophyllene (4.63%), limonene (3.76%), and *p*‐cymene (3.62%). These compounds have been associated with biological activities in tumor cell models, which may contribute to the effects observed on melanoma cell viability in the present study [1].

It is known that essential oils are rich in terpene compounds, such as monoterpenes and sesquiterpenes. In addition, extracts obtained with solvents such as ethanol and water can extract phenolic acids and polyphenols due to their chemical nature and the extraction processes used. These compounds possess bioactive properties, including antioxidant and anti‐inflammatory activities, which may contribute to the therapeutic effects of the extracts [29]. The observed effects may be associated with the presence of bioactive compounds such as terpenes, phenolic acids, and polyphenols, which have been previously reported in the literature for their biological activities [29].

Plants of the genus *Piper* are rich in lignans, a class of phenolic compounds [30, 31, 32]. These compounds have been reported to modulate biological responses in liver, colon, skin, oral, and breast cancer cell models, potentially acting directly or in synergy with traditional therapies to treat various types of cancer. In addition to their antitumor properties, lignans exhibit a range of biological activities, including antioxidant, anti‐inflammatory, antimicrobial, antiplatelet, neuroprotective, and antiparasitic effects [32].

### 3.3 Levels of Intracellular ROS, Nitrite/Nitrate, PSH, and NPSH

**FIGURE 3:**
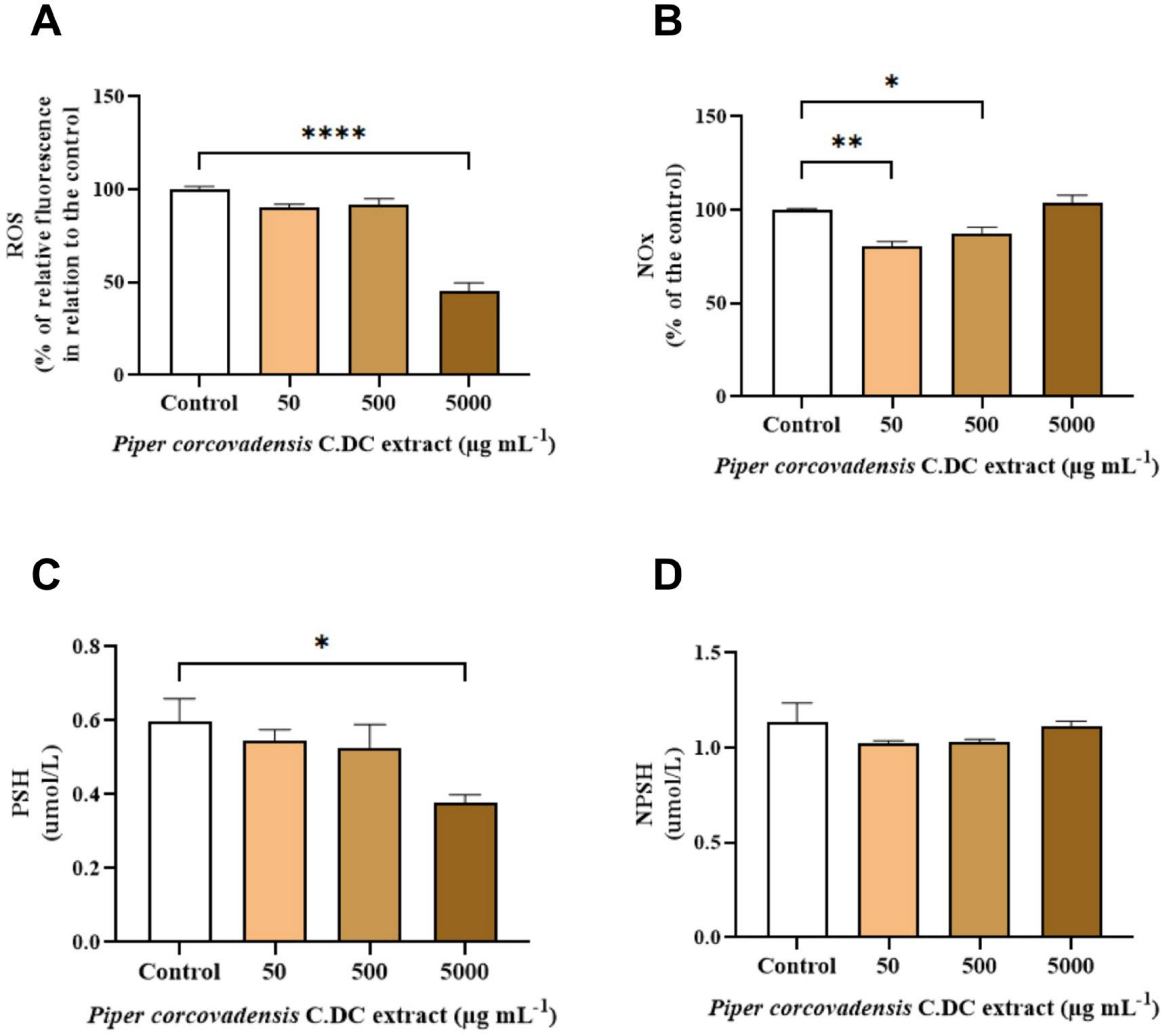
(A) Reactive oxygen species (ROS), (B) Nitric oxide (NOX), intracellular defenses: (C) Protein thiols (PSH) and (D) Non‐protein thiols (NPSH). Sample size (*n*): 3. Significance levels were defined by *p*‐values *(*p* < 0.05), **(*p* < 0.01), ****(*p* < 0.0001).

Analysis of ROS levels (Figure 3A) revealed a significant decrease at 5000 μg mL^−1^ compared to the control group (*p* < 0.0001). Regarding nitrite/nitrate levels (Figure 3B), a significant reduction was observed at a concentration of 50 μg mL^−1^ (*p* < 0.01) compared to the control. The nitrite/nitrate levels were also decreased at 500 μg mL^−1^ compared to the control (*p* < 0.05). However, at a concentration of 5000 μg mL^−1^, nitrite/nitrate levels did not differ significantly from the control (*p* > 0.05).

Regarding PSH (Figure 3C), there was a decrease at 5000 μg mL^−1^ compared to the control group (*p* < 0.05). However, at lower concentrations, there were no significant changes in this marker's levels. As for NPSH (Figure 3D) defenses, the applied treatments had no effect, with no reduction in levels compared to the control group (*p* > 0.05).

PSH (protein thiols) and NPSH (non‐protein thiols) are important components of cellular defenses, playing a crucial role in regulating the antioxidant response. They help protect cells against oxidative stress induced by ROS and/or NOX, contributing to the maintenance of redox homeostasis [33].

PSH (protein thiols) can be oxidized to PS‐SR (oxidized thiols or disulfides) in the presence of ROS and NOX, leading to a reduction in PSH levels, as well as in ROS and NOX levels. This process indicates the cell is experiencing oxidative stress [32].

The reduction in melanoma cell viability observed in this study may be associated with alterations in oxidative balance and inflammatory signaling; however, additional mechanistic studies are necessary to clarify the pathways involved.

### 3.4 Inflammatory Profile by RT‐qPCR

The inflammatory profile of *Piper corcovadensis* was assessed by analyzing the gene expression of *NLRP3* (NOD‐like receptor family, pyrin domain‐containing 3), *IL‐6* (Interleukin‐6), and TNF (Tumor Necrosis Factor). The study utilized a *Piper corcovadensis* concentration of 5000 μg mL^−1^, which resulted in the most significant decrease in SK‐MEL‐28 cell viability.

**FIGURE 4:**
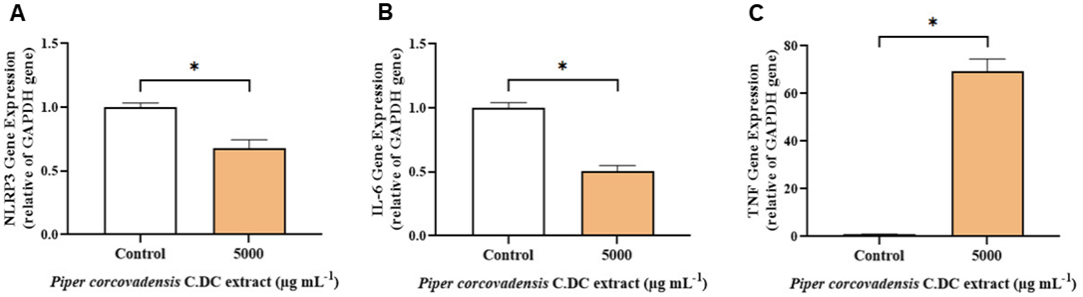
Evaluation of the inflammatory profile of *Piper corcovadensis* by RT‐qPCR, using the genes NLRP3 (NOD‐like receptor family, pyrin domain containing 3) (A), IL‐6 (Interleukin 6) (B), and TNF (Tumor Necrosis Factor) (C). Sample size (*n*): 4. Significance levels were defined by *p*‐values *(*p <* 0.05).

*Piper corcovadensis* treatment significantly downregulated *NLRP3* expression compared to the control group (Figure 4A, *p* < 0.05), suggesting a suppression of inflammasome activation. Similarly, *IL‐6* expression was reduced following *Piper corcovadensis* treatment (Figure 4B, *p* < 0.05). Conversely, *TNF* expression increased relative to the control (Figure 4C, *p* < 0.05), indicating modulation of inflammatory signaling pathways under the experimental conditions evaluated.

The activation of the *NLRP*3 gene initiates intracellular signaling pathways that drive inflammatory responses through cytokine production. The activation of the inflammasome has been linked to various inflammatory disorders and inflammation‐associated diseases [34]. *NLRP*3 activation plays a key role in inducing IL‐6 expression, which encodes a pro‐inflammatory cytokine critical to the immune response [23].

The *IL*‐6 gene is closely associated with inflammatory processes, as it drives the production of pro‐inflammatory cytokines secreted by immune cells in response to inflammation. Furthermore, *IL‐6* can suppress anti‐tumor immune responses, creating a microenvironment that favors tumor cell proliferation. Its activity is also influenced by the presence of ROS and NOX, further highlighting its role in inflammation and disease progression [35].

The *TNF* gene encodes a cytokine that plays a key role in physiological processes related to inflammation and cell death. This cytokine is also linked to autoimmune and neuroimmune disorders. Its expression leads to increased production of TNF‐α, which can stimulate inflammatory responses, regulate other cytokines such as *IL‐6*, and induce apoptosis [36].

*Piper corcovadensis* treatment reduced the expression of *NLRP3* and *IL‐6*, which converges to decreased ROS and NOX levels, both of which are known to trigger inflammatory processes. However, *Piper corcovadensis* increased *TNF* expression at 5000 μg mL^−1^, the same concentration at which low cell viability was observed. The modulation of TNF expression may be associated with the reduction in SK‐MEL‐28 viability observed in the present study.

### 3.5 Antibacterial Activity

The antibacterial activity of *Piper corcovadensis* extract was evaluated against pathogenic microorganisms associated with foodborne infections. The results are presented in Table 2.

**TABLE 2:** Minimum inhibitory concentration (MIC) and minimum bactericidal concentration (MBC) of *Piper corcovadensis*.

Microorganisms	MIC (mg mL^−1^)	MBC (mg mL^−1^)
*Bacillus subtilis* ^(+)^	> 5	> 5
*Listeria monocytogenes* ^(+)^	> 5	> 5
*Escherichia coli* ^(−)^	> 5	> 5
*Salmonela Typhimurium* ^(−)^	> 5	> 5

*Note:* ^(+)^gram positive microorganism; ^(−)^gram‐negative microorganism.

This analysis aimed to determine the concentration at which the phenolic compounds in *Piper corcovadensis* inhibit protein and nucleic acid synthesis or interfere with essential physiological processes, thereby defining the minimum inhibitory concentration (MIC). Additionally, the minimum bactericidal concentration (MBC) is the concentration required to disrupt the bacterial cell wall, leading to leakage of cellular contents and cell death [37].

For all tested bacteria, both the MIC and MBC were higher than 5 mg mL^−1^, suggesting that the effect of *Piper corcovadensis* is not fully observed within the tested concentration range. Regarding these findings, higher concentrations of the *Piper corcovadensis* extract are necessary to stop the growth of all bacteria tested in this study.

However, the plant extracts are rich in compounds derived from the specialized plant metabolism, including alkaloids, terpenes, phenolic acids, and flavonoids, which have demonstrated antimicrobial properties against a broad spectrum of pathogens [36].

Studies on *Piper nigrum* have shown that its ethanolic extract can induce bacterial cell death, with MIC and MBC values of 1250 μg mL^−1^ for Gram‐positive bacteria and 156.25 μg mL^−1^ for Gram‐negative bacteria [37]. These findings suggest that antimicrobial effects may be observed at higher concentrations, considering the chemical composition of the species [37].

Similarly, the *Piper rivinoides* exhibited antifungal activity against *Candida tropicalis* and *Candida albicans* , with enhanced efficacy when combined with antifungal drugs, reducing the required drug concentration [38]. Furthermore, studies on *Piper regenelli* demonstrated antibacterial potential against *Staphylococcus aureus* and antifungal activity against *Candida tropicalis* and *Candida albicans* , contributing to reduced microbial virulence and resistance [39, 40].

## 4 Conclusion

The findings of this study suggest that *Piper corcovadensis* extract exhibits in vitro antioxidant activity, modulates oxidative stress and inflammatory markers, and affects melanoma cell viability under the experimental conditions evaluated. Regarding cytotoxicity, the extract reduced SK‐MEL‐28 cell viability in a concentration‐dependent manner, with no significant toxicity observed in PBMCs. However, it is important to emphasize that the concentrations required to induce significant effects were relatively high, which is commonly observed in studies using crude plant extracts and highlights the need for further fractionation and identification of active compounds.

The modulation of oxidative stress markers revealed a decrease in ROS and NOx levels, accompanied by alterations in thiol‐based defenses. These findings suggest that the extract may interfere with cellular redox homeostasis. Additionally, the modulation of NLRP3, IL‐6, and TNF expression suggests an interaction with inflammatory signaling pathways that may be associated with the observed reduction in melanoma cell viability.

The extracts of *Piper corcovadensis* showed no antibacterial activity against all bacteria tested and did not allow determination of MIC and MBC values within the tested concentration range.

Further studies involving compound isolation, mechanistic assays, and in vivo approaches are necessary to better understand the biological effects and potential applications of this species.

## Author Contributions

B.H.F. and E.C.P.: investigation, writing original draft, formal analysis, data curating. M.D.B.: supervision, resources, data curating. D.M.: formal analysis, data curating. G.B.S.: formal analysis, data curating. M.F.F.R.: supervision, resources, data curating. J.A.M.: supervision, resources, data curating. S.T.C.: project administration, writing, review and editing, supervision, resources, data curating. All authors were involved in drafting and revising the manuscript and approved the final manuscript.

## Funding

This study was funded by grants from Conselho Nacional de Desenvolvimento Científico e Tecnológico (CNPq): BHF (#140275/2024‐0) and Instituto Nacional Sinalização Purinérgica: Desafios para a saúde do século 21; N° (409156/2024–8), MDB; (305974/2025–4), Fundação de Amparo à Pesquisa e Inovação do Estado de Santa Catarina (FAPESC) (MDB; 2023TR001472, 2024TR002502, 2025TR001686) and Federal University of Fronteira Sul. MDB is a CNPq Research Fellow. The authors thank the Coordination for the Improvement of Higher Education Personnel (CAPES) for postgraduate scholarships.

## Ethics Statement

The authors have nothing to report.

## Consent

The authors have nothing to report.

## Conflicts of Interest

The authors declare no conflicts of interest.

## Data Availability

The datasets used and/or analyzed during the current study are available from the corresponding author on reasonable request.
